# Supplementary material for: Discovery and remodeling of Vibrio natriegens as a microbial platform for efficient formic acid biorefinery
Source: Nat Commun. 2023 Nov 27;14:7758. doi: 10.1038/s41467-023-43631-2 (PMC10682008; doi:10.1038/s41467-023-43631-2)
Supplement: Supplementary file 1 — Supplementary information [file 41467_2023_43631_MOESM1_ESM.pdf]

Discovery and remodeling of *Vibrio natriegens* as a microbial platform for efficient formic acid biorefinery

Tian *et al*.

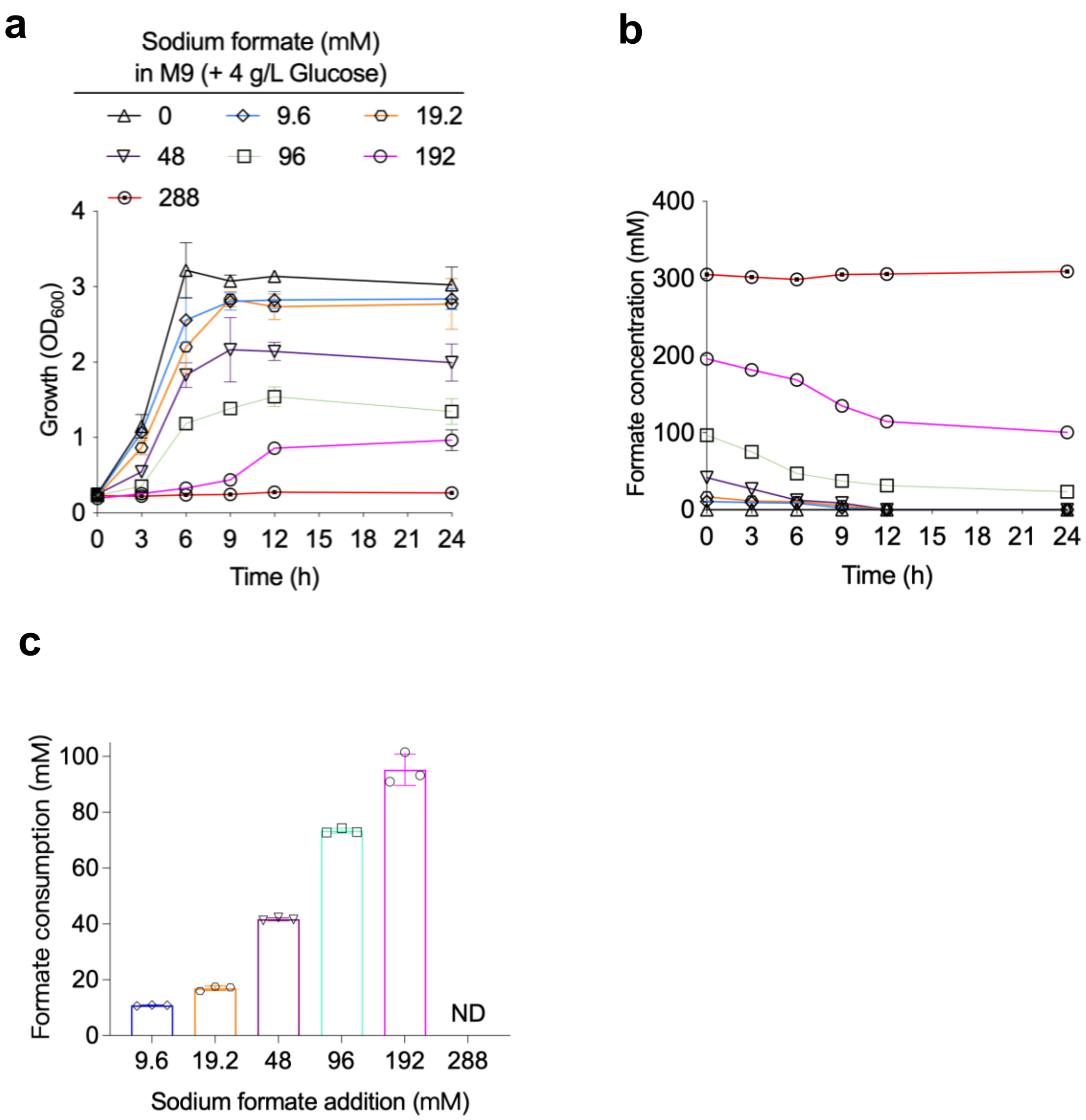

Supplementary Figure 1. Formate tolerance and consumption of the *V. natriegens* wild-type strain grown in the M9 minimal medium. a, The growth of *V. natriegens* in the M9 medium (containing 4 g·L^{-1} glucose) with varying formate concentrations. **b**, Residual formate concentrations after 24 h cultivation of *V. natriegens* with different initial concentrations of formate. **c**, The formate consumption of *V. natriegens* after 24 h cultivation with initial supplementation of different amounts of sodium formate. Data are presented as the mean ± SD (*n* = 3 biologically independent samples). Error bars show SDs. Source data are provided as a Source Data file.

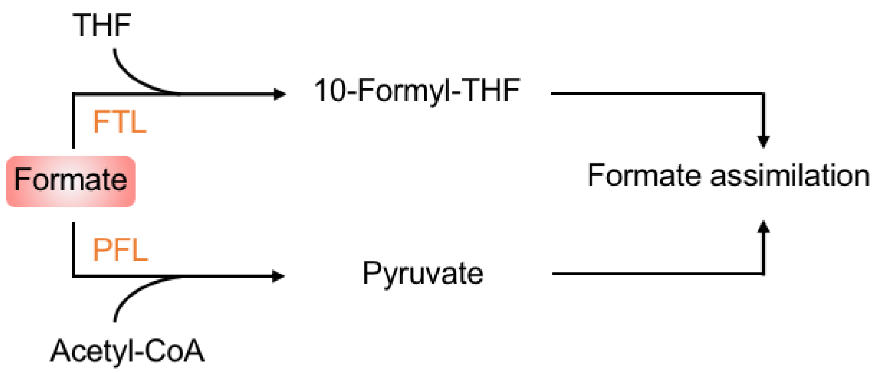

Supplementary Figure 2. FTL and PFL-mediated formate assimilation reactions in microorganisms. FTL, formate tetrahydrofolate ligase. PFL, pyruvate formate-lyase.

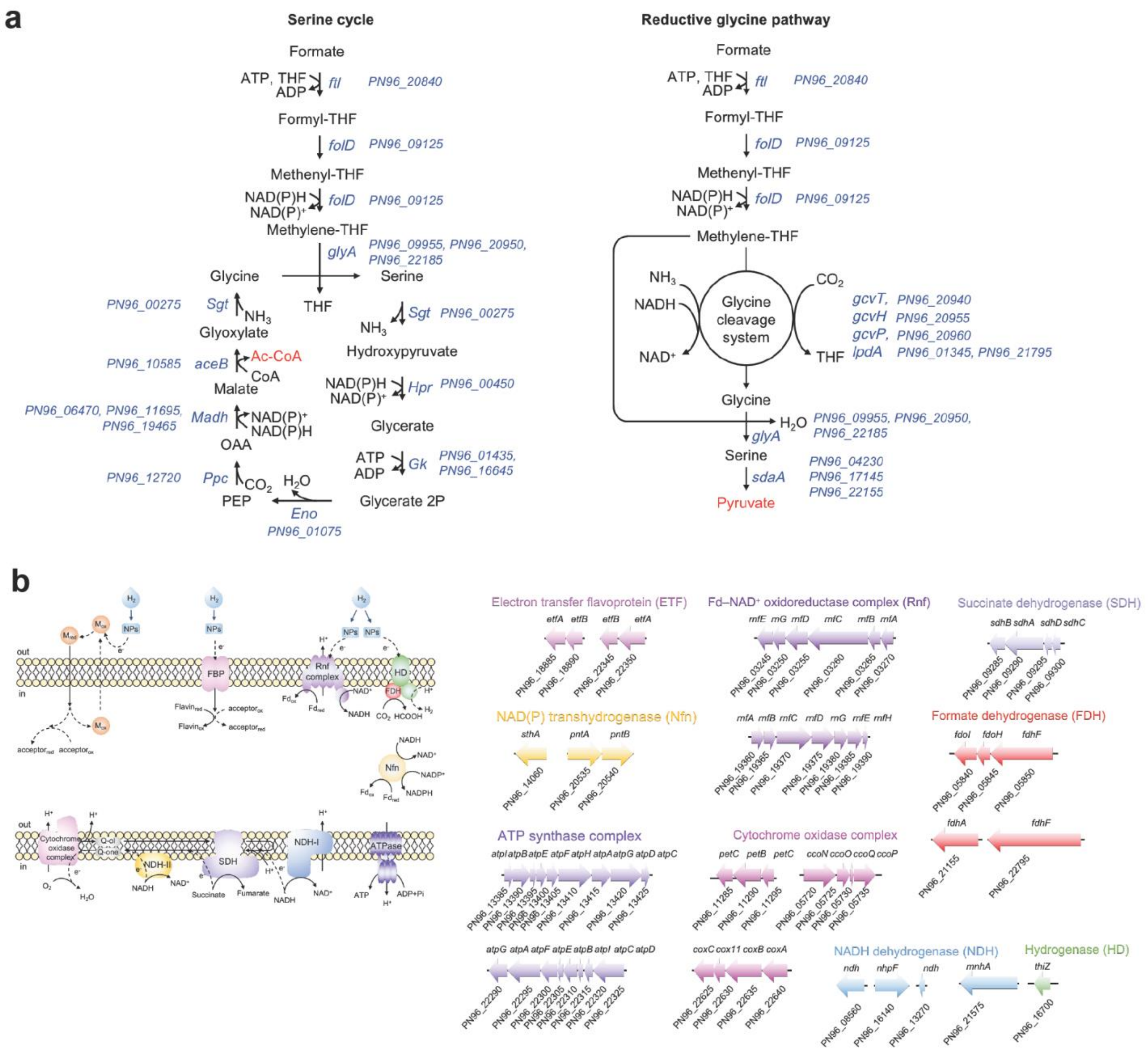

Supplementary Figure 3. The annotated genes associated with the serine cycle, reductive glycine pathway, and energy conservation and supply systems in *V. natriegens*. a, The genes responsible for the serine cycle and reductive glycine pathway in *V. natriegens*. **b**, Putative energy conservation and supply systems in *V. natriegens* and arrangement of the associated genes in the genome. They include metal ion mediated EET (extracellular electron transfer), flavin binding protein (FBP), Fd-NAD$^+$ oxidoreductase complex (Rnf), Hydrogenase (HD), formate dehydrogenase (FDH), transhydrogenase (Nfn), Cytochrome oxidase complex, NADH dehydrogenase II (NDH-II), succinate dehydrogenase (SDH), NADH dehydrogenase I (NDH-I), ATPase (ATP synthase), metal ion (M) and nano particles (NPs).

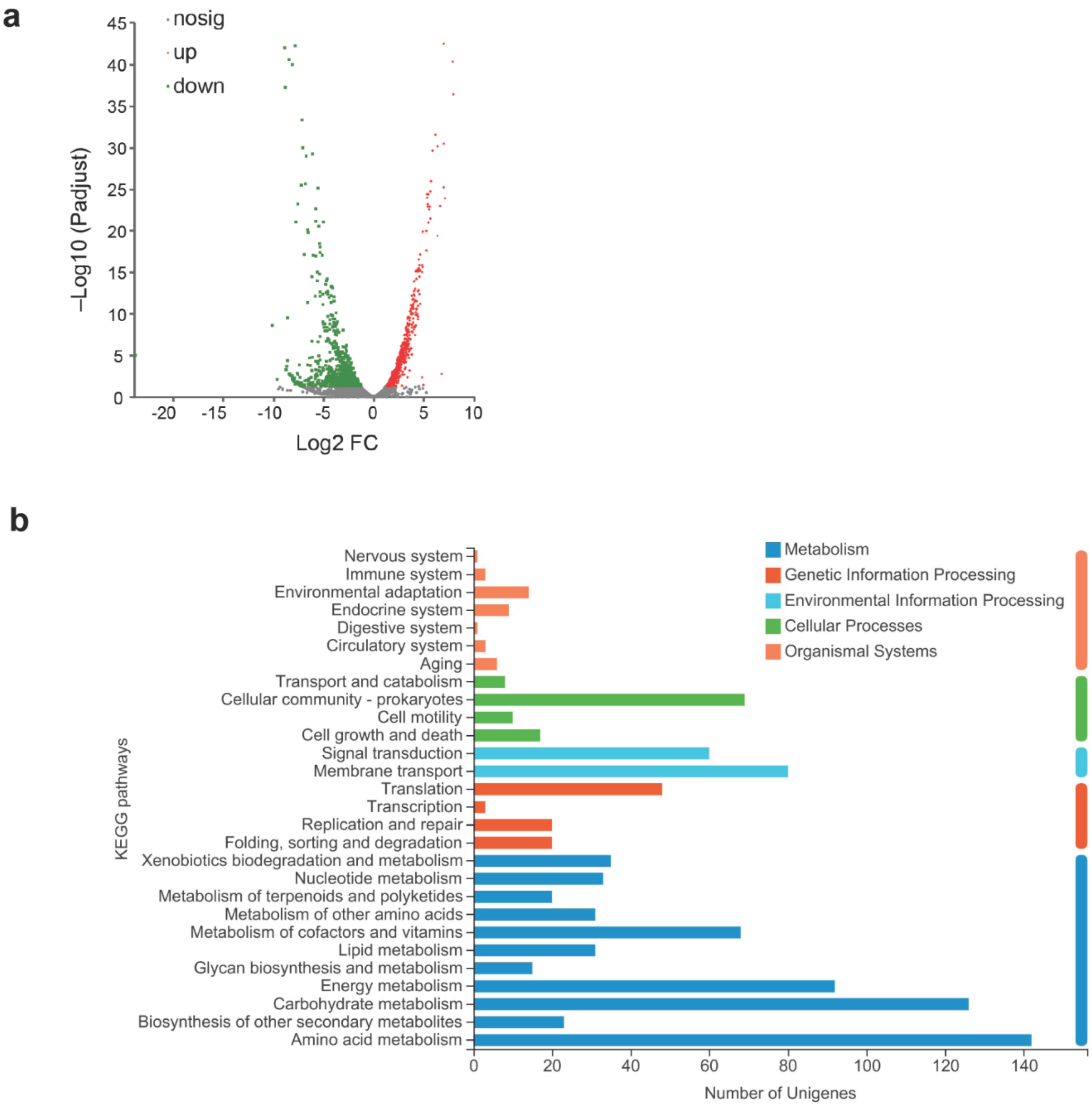

Supplementary Figure 4. Comparative transcriptomic analysis of *V. natriegens* in the presence and absence of formate using RNA-seq. a, Volcano map of differentially expressed genes. Each dot represents one gene. The red and green dots indicate the genes exhibiting significantly upregulation and downregulation (FDR ≤ 0.05 and $|log_2FC| \geq 1$), respectively, in the presence of 40 $g \cdot L^{-1}$ sodium formate ($HCOONa \cdot 2H_2O$). The other genes were represented by grey dots. **b**, The top 28 functional enrichment subsets generated from the data of comparative transcriptomic analysis.

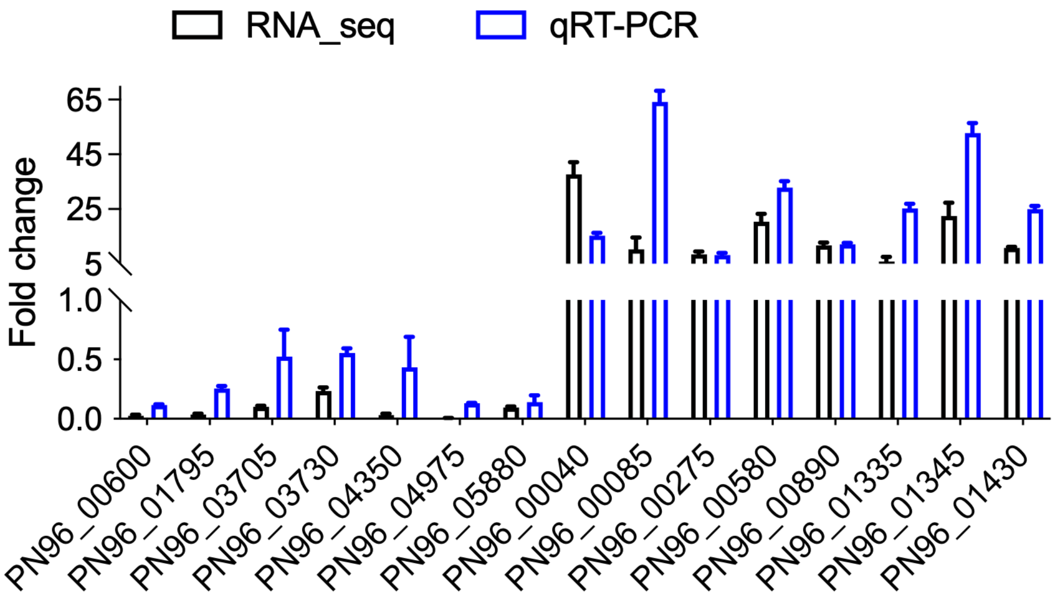

Supplementary Figure 5. Correlation between the RNA-seq and qRT-PCR results. The *V. natriegens* strains were cultured in the LBv2 medium containing 40 g L^{-1} sodium formate ($HCOONa \cdot 2H_2O$). Cells were harvested after 6 h of fermentation. 15 genes that showed significantly changed transcriptional levels with formate stress (RNA-seq results) were picked out for testing. The RNA_seq data were mean ± standard deviation (SD) of two independent biological replicates. The qRT-PCR data were mean ± standard deviation (SD) of three independent biological replicates. Error bars show SDs. Source data are provided as a Source Data file.

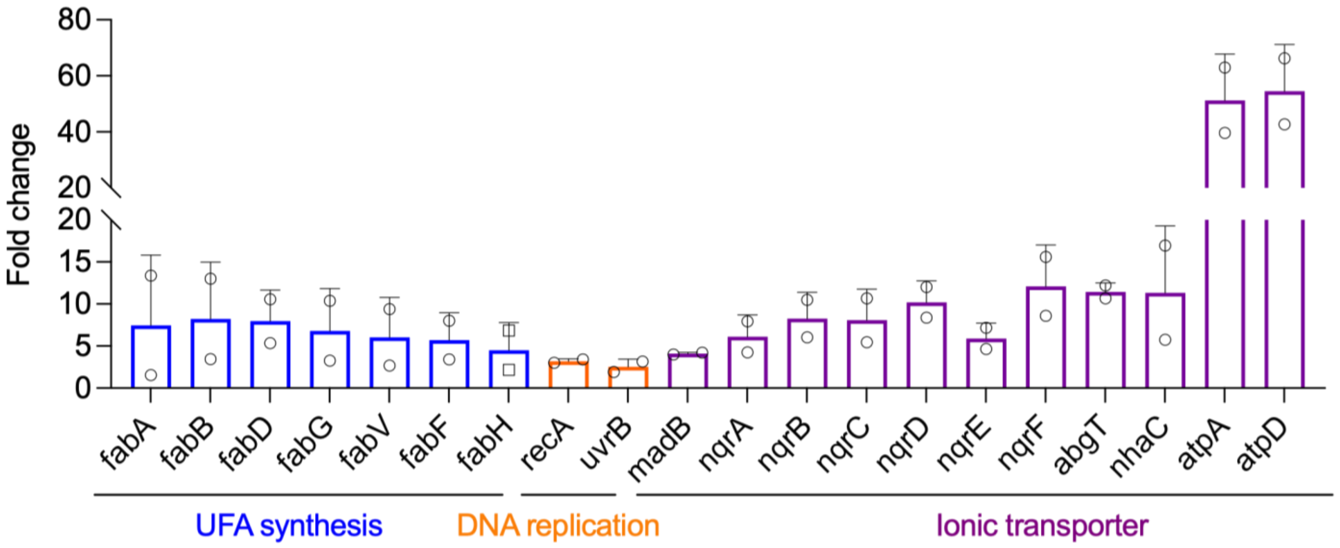

Supplementary Figure 6. Transcriptional changes of the *V. natriegens* genes which are responsible for ionic efflux systems, synthesis of unsaturated fatty acids, and DNA repairing with the stress of sodium formate. The data were mean ± standard deviation (SD) of two independent biological replicates. Error bars show SDs.

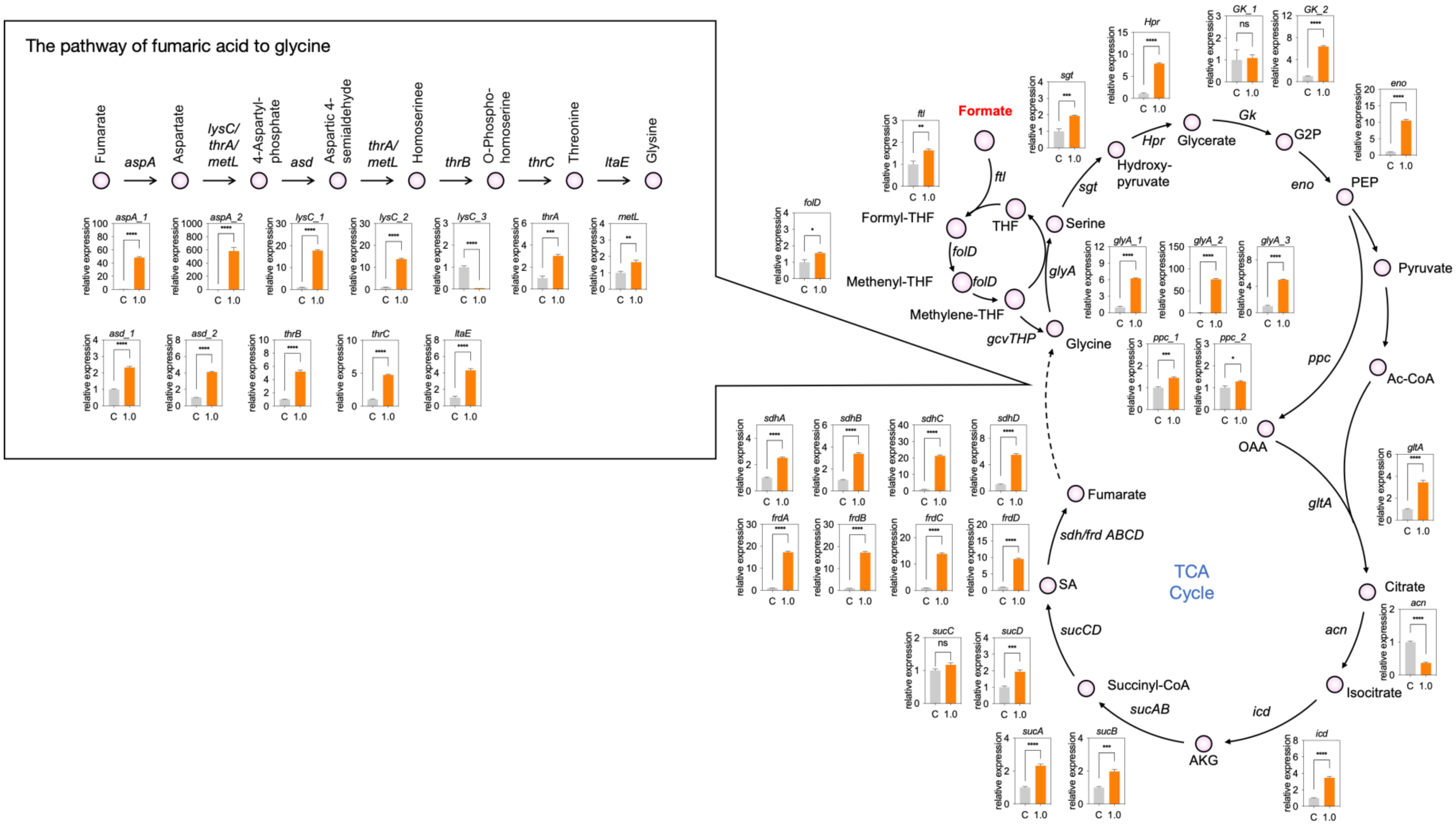

Supplementary Figure 7. Transcriptional difference of the S-TCA genes between the wild-type and S-TCA-1.0 strains. The LBv2 medium was used with the supplementation of 40 g·L^{-1} sodium formate ($HCOONa \cdot 2H_2O$). The samples for the real-time qRT-PCR analysis were collected after 12 h of cultivation. C: the control strain (wild-type). 1.0: the S-TCA-1.0 strain. Data are presented as the mean ± SD (n = 4 biologically independent samples). Error bars show SDs. Statistical analysis was performed by a two-tailed Student's t-test. *, $P < 0.05$; **, $P < 0.01$; ***, $P < 0.001$; ****, $P < 0.0001$ versus the control strain. Source data are provided as a Source Data file.

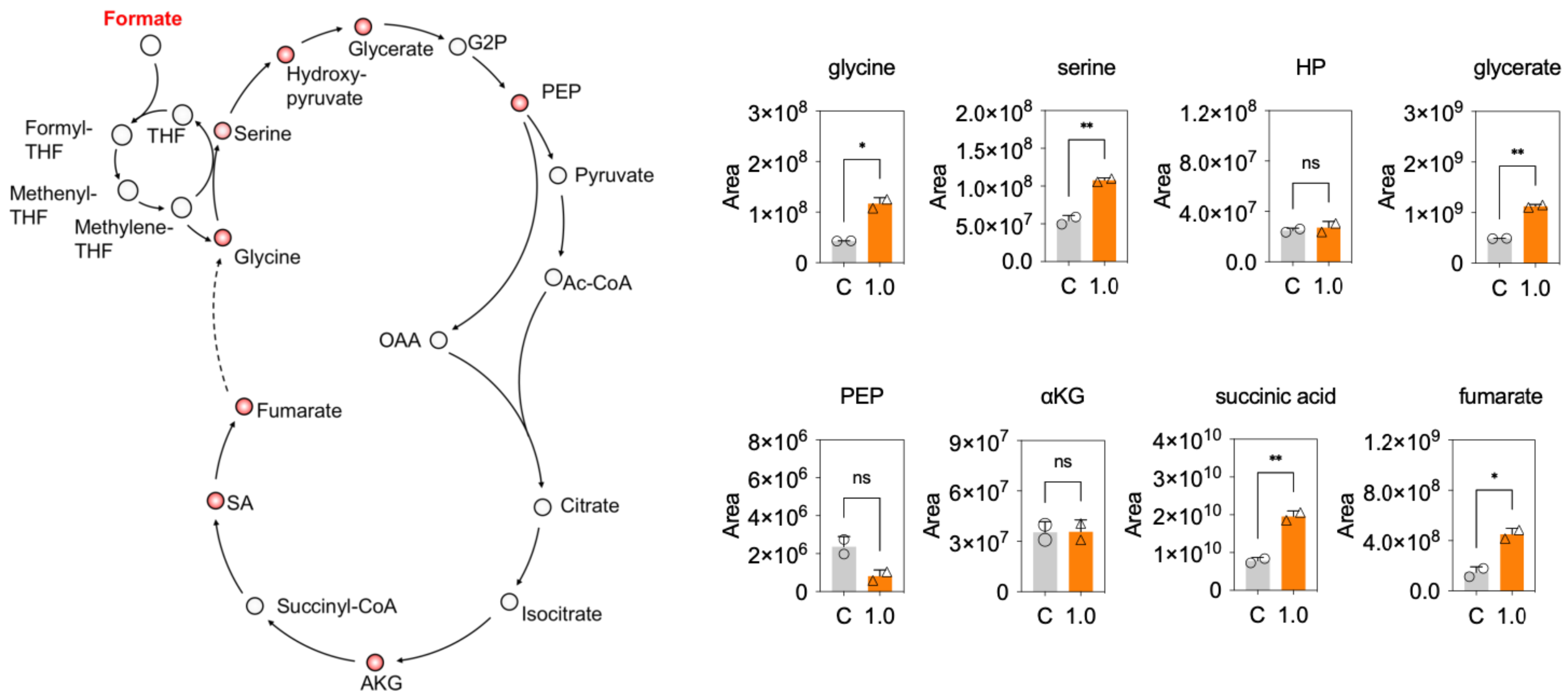

Supplementary Figure 8. Differences of metabolite levels in the S-TCA cycle between the S-TCA-1.0 and the wild-type strains. The LBv2 medium was used with the supplementation of 40 g·L^{-1} sodium formate ($HCOONa \cdot 2H_2O$). HP: hydroxypyruvate. α-KG: α-ketoglutarate. C: the control strain (wild-type). 1.0: the S-TCA-1.0 strain. Data are presented as the mean ± SD (n = 2 biologically independent samples). Error bars show SDs. Statistical analysis was performed by a two-tailed Student's t-test. *, $P < 0.05$; **, $P < 0.01$; ***, $P < 0.001$; versus the control strain. ns: no significance. Source data are provided as a Source Data file.

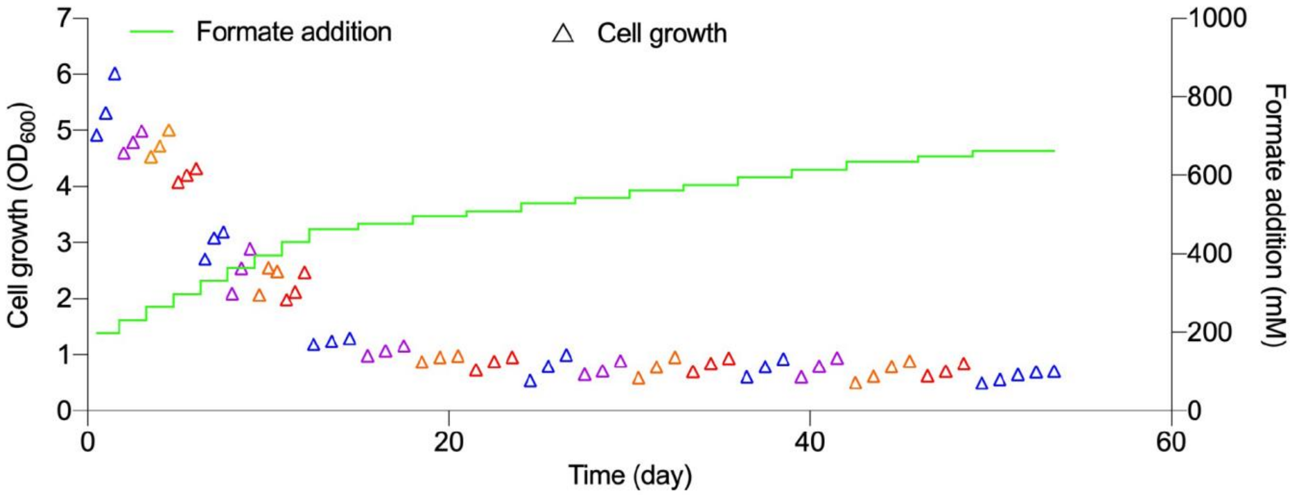

Supplementary Figure 9. The evolution trajectory of the wild-type *V. natriegens* strain for obtaining an evolved strain with enhanced formate utilization. The LBv2 medium was used with increasing concentrations of sodium formate ($HCOONa·2H_2O$) from 20 to 70 g·L^{-1} (the equivalent of 192 to 673 mM formate). Triangles indicate the bacterial biomass of each passage after 12 h of cultivation, in which same colors represent the passages with the same concentration of sodium formate. Source data are provided as a Source Data file.

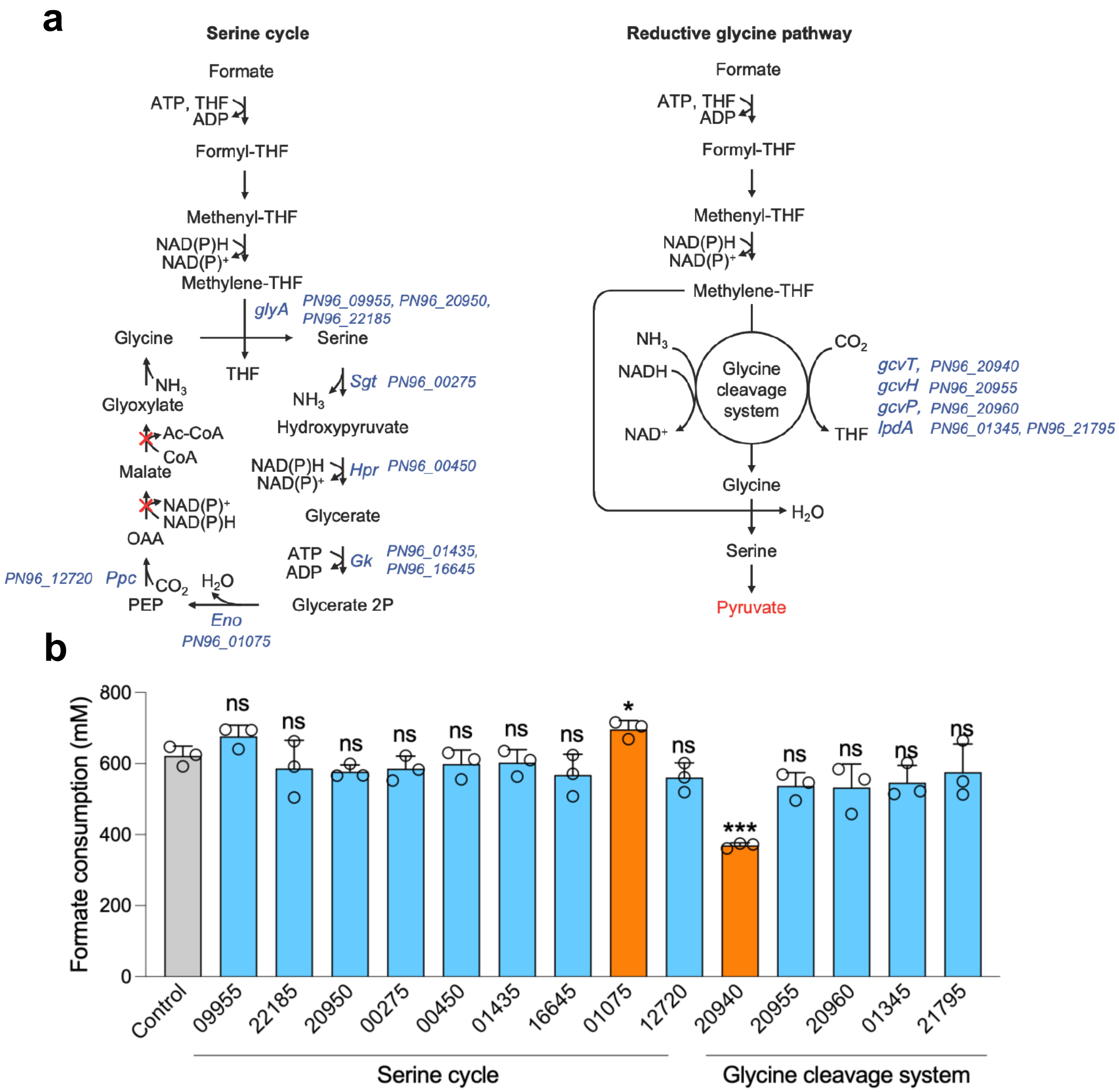

Supplementary Figure 10. Influence of overexpressing the genes of the glycine cleavage system (GCS) and the serine cycle on formate consumption of the S-TCA-2.0 strain. a, The overexpressed genes of GCS and the serine cycle in S-TCA-2.0. **b**, Influence of overexpressing these genes on the formate consumption of S-TCA-2.0. The LBv2 medium was used with the addition of 80 g·L^{-1} sodium formate ($HCOONa \cdot 2H_2O$). Data are presented as the mean ± SD (n = 3 biologically independent samples). Error bars show SDs. Statistical analysis was performed by a two-tailed Student's t-test. *, $P < 0.05$; ***, $P < 0.001$; versus the control strain. Source data are provided as a Source Data file.

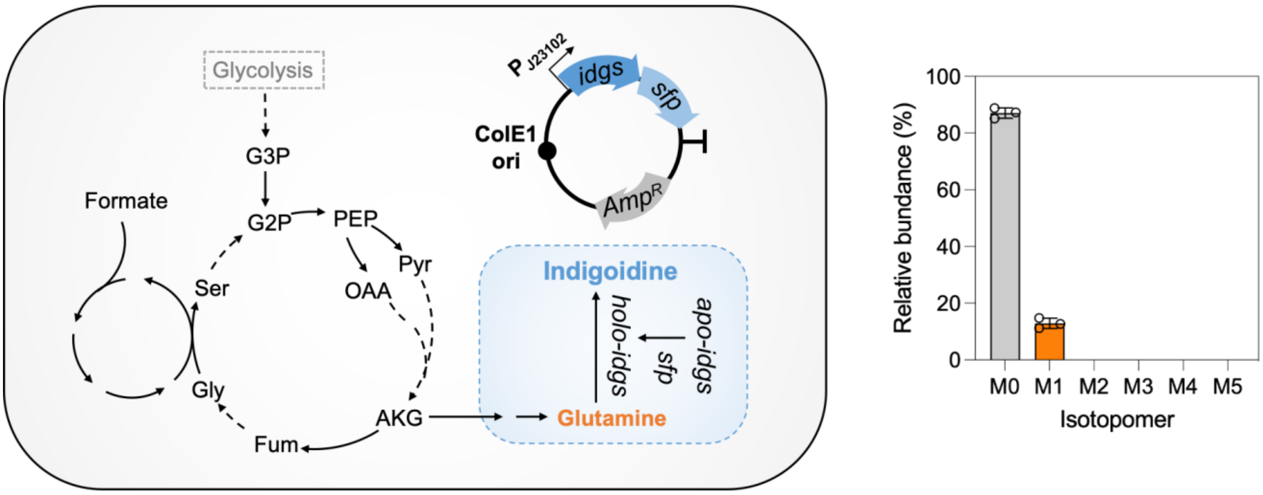

Supplementary Figure 11. Relative abundance of ^{13}C-labelled glutamine in the S-TCA-2.0-IE strain. The cells were grown in the LBv2 medium supplemented with 60 g·L^{-1} of ^{13}C-labelled sodium formate. Data are presented as the mean ± SD (*n* = 3 biologically independent samples). Error bars show SDs. Source data are provided as a Source Data file.

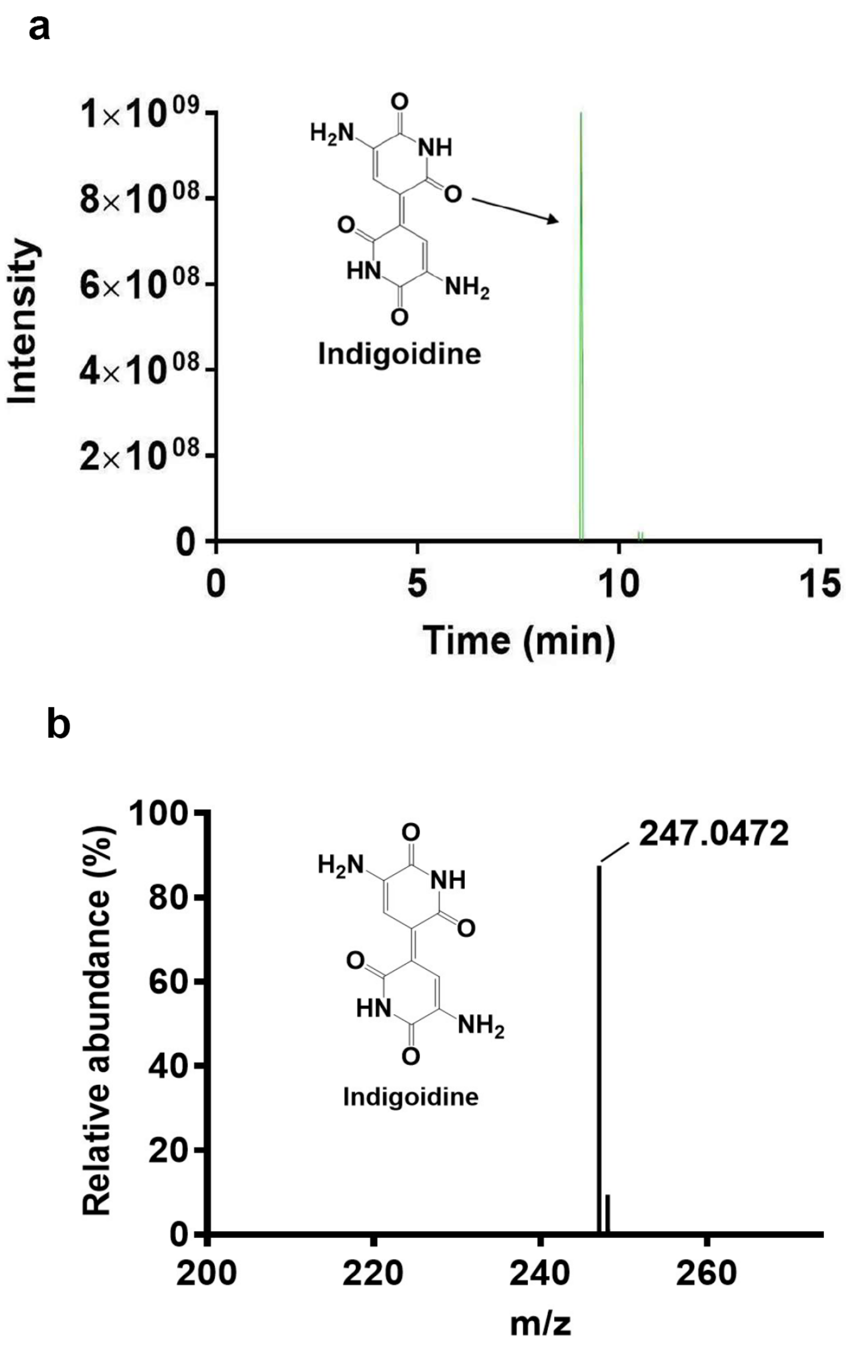

Supplementary Figure 12. High-performance liquid chromatography-mass spectrometry identification of the extracted indigoidine. a, Detection of the extracted indigoidine by HPLC. **b**, Mass spectrometry analysis of the HPLC fraction corresponding to indigoidine.

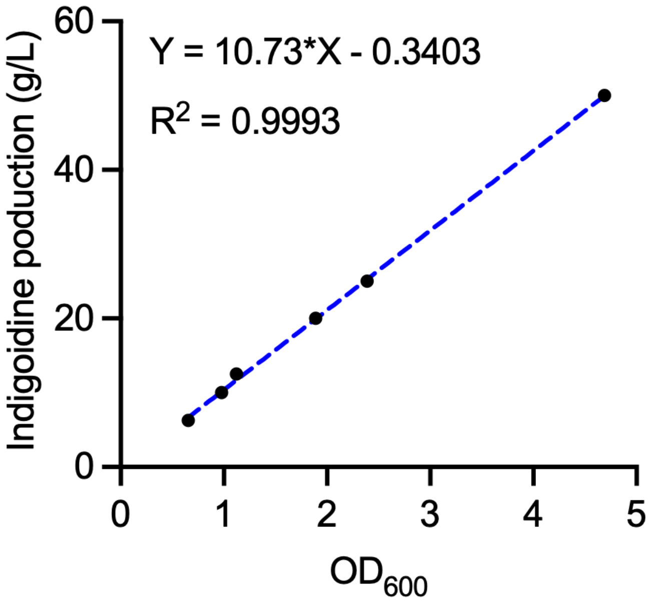

Supplementary Figure 13. Spectrophotometer standard curve correlating indigoidine concentration to the absorbance at 600 nm. Source data are provided as a Source Data file.

Supplementary Table 1. Comparison of the formate consumption of the *V. natriegens* strains and other microorganisms (some model microorganisms and reported formate-utilizing microorganisms).

Microorganisms	Working concentration of formate (mM)	Formate consumption rate ($mM\cdot h^{-1}$)	Carbon sources	References
Vibrio natriegens	385	11.92	Formate, yeast extract, peptone	This study
	192	3.96	Formate, glucose	This study
Vibrio natriegens S-TCA-2.0*	817	31.6	Formate, yeast extract, peptone	This study
Saccharomyces cerevisiae	250	0	Formate, glucose, CO_2	1
Umbelopsis isabellina	88.9	1.86	Formate, yeast extract, glucose	2
Thermococcus onnurineus	111.1	ND	Formate	3
Thermoacidophilic crenarchaeote	0.05	0.00017	Formate	4
Rhodopseudomonas palustris	3.33	2.79	Formate	5
Ralstonia eutropha	43.5	0.95	Formate	6
Pseudomonas C	109	ND	Formate	7
Kuenenia stuttgartiensis	50	ND	Formate, acetate, CO_2	8
Desulfurococcus amylolyticus	116.6	0.017	Formate	9
Desulfovibrio desulfuricans	75	ND	Formate, CO_2	10
Clostridium pasteurianum	117.6	0	Formate, yeast extract, glucose	11
Methanothermobacter spp.	200	3.95	Formate	12
Dehalococcoides mccartyi	2	ND	Formate, acetate	13
Acetobacterium wieringae	1	ND	Formate, CO	14
*Escherichia coli**	86.9	4.04	Formate, CO_2, glucose	15
*Escherichia coli**	86.9	2.89	Formate, CO_2	16
*Escherichia coli**	60	0.357	Formate, CO_2	17

*Artificial bacteria. ND, not detected.

Supplementary Table 2. Specific glucose, glycine, and formate consumption rates and ^{13}C ratio in biomass of the V. natriegens S-TCA-2.0 strain.

Samples	Specific glucose consumption rate ($mg \cdot gDCW^{-1} \cdot h^{-1}$)	Specific glycine consumption rate ($mg \cdot gDCW^{-1} \cdot h^{-1}$)	Specific formate consumption rate ($mg \cdot gDCW^{-1} \cdot h^{-1}$)	^{13}C ratio in biomass* (mol/mol)	Specific formate assimilation rate§ ($mg \cdot gDCW^{-1} \cdot h^{-1}$)
No.1	292.4	53.6	379.8	0.078	42.7 (11.2%)
No.2	292.4	44.6	335.4	0.082	43.9 (13.0%)
Average	292.4	49.1 ± 4.5	357.6 ± 22.2	0.080 ± 0.002	43.3 ± 0.6 (12.1% ± 0.9%)

*The assay of ^{13}C ratio in biomass was performed according to the protocol in Methods section.

§The specific formate assimilation rate is not a measured value. It is calculated based on the following formula referring to the previous report[15]: (^{13}C ratio in biomass) = (assimilated carbon moles from formate)/(assimilated carbon moles from formate, glucose, and glycine). Here, the consumed glucose and glycine were assumed to be completely assimilated. The number in the brace is calculated by (specific formate assimilation rate)/(specific formate consumption rate), indicating how much formate was assimilated into biomass.

Supplementary Table 3. The reported indigoidine production through microbial synthesis.

Microorganisms	Titer ($g \cdot L^{-1}$)	Productivity ($g \cdot L^{-1} \cdot h^{-1}$)	Carbon sources	Fermentation mode	References
Escherichia coli	1.73 (25 ℃)<br>2.78 (18 ℃)	0.133 (25 ℃)<br>0.099 (18 ℃)	Yeast extract, peptone	Batch	18
Streptomyces coelicolor	0.59	0.004	Sucrose, glucose, yeast extract	Batch	18
Escherichia coli	14	0.25	Yeast extract, glutamate	Batch	19
Pseudomonas putida	25.6	0.22	Glucose	Fed-batch	20
Escherichia coli	8.81	0.315	Yeast extract, peptone, glutamine	Batch	21
Saccharomyces cerevisiae	0.98	0.014	Yeast extract, peptone, glucose	Fed-batch	22

Supplementary Table 4. Strains and plasmids used in this work.

Strains/plasmids	Description/genotype	Sources
**Strains**		
E. coli DH5α	For plasmid construction	Invitrogen
V. natriegens ATCC 14048	The parental *V. natriegens* strain	23
S-TCA-1.0	Derived from *V. natriegens* ATCC 14048, Δ*PN96_00930*Δ*PN96_07295*Δ*PN96_10585*Δ*PN96_14755*Δ*PN96_06470*Δ*PN96_19465*Δ*PN96_11695::Km*^{R}Δ*dns::Cm*^{R}	This study
S-TCA-2.0	Derived from S-TCA-1.0 by adaptive laboratory evolution	This study
S-TCA-2.0-IE	Derived from S-TCA-2.0 for indigoidine production	This study
V. natriegens Δ*fdh*	Derived from *V. natriegens* ATCC 14048, with simultaneous deletion of six *fdh* genes (PN96_05840, PN96_05845, PN96_05850, PN96_05880, PN96_21155, and PN96_22795)	This study
V. natriegens Δ*ftl*	Derived from *V. natriegens* ATCC 14048, with deletion of the *ftl* gene (PN96_20840)	This study
V. natriegens Δ*pfl*	Derived from *V. natriegens* ATCC 14048, with deletion of the *pfl* gene (PN96_08455)	This study
V. natriegens Δ*fdh*Δ*ftl*Δ*pfl*	Derived from *V. natriegens* ATCC 14048, with simultaneous deletion of *ftl* (PN96_20840), *pfl* (PN96_08455), and six *fdh* genes (PN96_05840, PN96_05845, PN96_05850, PN96_05880, PN96_21155, and PN96_22795).	This study
**Plasmids**		
pColE1-Amp	Overexpression plasmid with pColE1 origin, ampicillin/carbenicillin resistance	24
pColE1-idgs-sfp	Derived from pColE1-Amp, carrying the DNA fragment of J23102-B00320m-idgs-RBS-sfp	This study
pMMB67EH-tfox	The IPTG-inducible plasmid for expressing *tfoX*, ampicillin/carbenicillin resistance, pMMB origin	25
pMMB67EH-tfox-SacB	Derived from pMMB67EH-tfox, for *SacB* overexpression	This study

Supplementary references

1. Gonzalez de la Cruz, J., Machens, F., Messerschmidt, K. & Bar-Even, A. Core catalysis of the reductive glycine pathway demonstrated in Yeast. *ACS Synth. Biol.* **8**, 911-917 (2019).
2. Liu, Z. *et al*. Exploring eukaryotic formate metabolisms to enhance microbial growth and lipid accumulation. *Biotechnol. Biofuels* **10**, 22 (2017).
3. Moon, Y. J. *et al.* Proteome analyses of hydrogen-producing hyperthermophilic archaeon *Thermococcus onnurineus* NA1 in different one-carbon substrate culture conditions. *Mol. Cell. Proteomics* **11**, M111.015420 (2012).
4. Urschel, M. R., Hamilton, T. L., Roden, E. E. & Boyd, E. S. Substrate preference, uptake kinetics and bioenergetics in a facultatively autotrophic, thermoacidophilic crenarchaeote. *FEMS. Microbiol. Ecol.* **92**, fiw069 (2016).
5. Stokes, J. E. & Hoare, D. S. Reductive pentose cycle and formate assimilation in *Rhodopseudomonas palustris. J. Bacteriol.* **100**, 890-894 (1969).
6. Grunwald, S. *et al*. Kinetic and stoichiometric characterization of organoautotrophic growth of *Ralstonia eutropha* on formic acid in fed-batch and continuous cultures. *Microb. Biotechnol.* **8**, 155-163 (2015).
7. Goldberg, I. & Mateles, R. I. Growth of *Pseudomonas C* on C1 compounds: enzyme activites in extracts of *Pseudomonas C* cells grown on methanol, formaldehyde, and formate as sole carbon sources. *J. Bacteriol.* **122**, 47-53 (1975).
8. Lawson, C. E. *et al*. Autotrophic and mixotrophic metabolism of an anammox bacterium revealed by in vivo ^{13}C and ^{2}H metabolic network mapping. *ISME J.* **15**, 673-687 (2021).
9. Ergal, I. *et al*. Formate utilization by the crenarchaeon *Desulfurococcus amylolyticus*. *Microorganisms* **8**, 454 (2020).
10. Sánchez-Andrea, I. *et al*. The reductive glycine pathway allows autotrophic growth of *Desulfovibrio desulfuricans*. *Nat. Commun.* **11**, 5090 (2020).
11. Hong, Y., Arbter, P., Wang, W., Rojas, L. N. & Zeng, A. P. Introduction of glycine synthase enables uptake of exogenous formate and strongly impacts the metabolism in *Clostridium pasteurianum*. *Biotechnol. Bioeng.* **118**, 1366-1380 (2021).
12. Fink, C. *et al*. A shuttle-vector system allows heterologous gene expression in the thermophilic methanogen *Methanothermobacter thermautotrophicus* ΔH. *mBio.* **12**, e0276621 (2021).
13. Zhuang, W. Q. *et al*. Incomplete Wood-Ljungdahl pathway facilitates one-carbon metabolism in organohalide-respiring *Dehalococcoides mccartyi*. *Proc. Natl Acad. Sci. USA*

111, 6419-6424 (2014).

14. Arantes, A. L. *et al*. Enrichment of anaerobic syngas-converting communities and isolation of a novel carboxydotrophic *Acetobacterium wieringae* strain JM. *Front. Microbiol.* **11**, 58 (2020).
15. Bang, J. & Lee, S. Y. Assimilation of formic acid and CO_2 by engineered *Escherichia coli* equipped with reconstructed one-carbon assimilation pathways. *Proc. Natl Acad. Sci. USA.* **115**, E9271-E9279 (2018).
16. Bang, J., Hwang, C. H., Ahn, J. H., Lee, J. A. & Lee, S. Y. *Escherichia coli* is engineered to grow on CO_2 and formic acid. *Nat. Microbiol.* **5**, 1459-1463 (2020).
17. Kim, S. *et al*. Growth of *E. coli* on formate and methanol via the reductive glycine pathway. *Nat. Chem. Biol.* **16**, 538-545 (2020).
18. Yu, D., Xu, F., Valiente, J., Wang, S. & Zhan, J. An indigoidine biosynthetic gene cluster from *Streptomyces chromofuscus* ATCC 49982 contains an unusual IndB homologue. *J. Ind. Microbiol. Biotechnol.* **40**, 159-168 (2013).
19. Wang, L. *et al*. Protein scaffold optimizes arrangement of constituent enzymes in indigoidine synthetic pathway to improve the pigment production. *Appl. Microbiol. Biotechnol.* **104**, 10493-10502 (2020).
20. Banerjee, D. *et al*. Genome-scale metabolic rewiring improves titers rates and yields of the non-native product indigoidine at scale. *Nat. Commun.* **11**, 5385 (2020).
21. Xu, F., Gage, D. & Zhan, J. Efficient production of indigoidine in *Escherichia coli*. *J. Ind. Microbiol. Biotechnol.* **42**, 1149-1155 (2015).
22. Wehrs, M. *et al*. Correction to: Production efficiency of the bacterial non-ribosomal peptide indigoidine relies on the respiratory metabolic state in *S. cerevisiae*. *Microb. Cell Fact.* **18**, 218 (2019).
23. Weinstock, M. T., Hesek, E. D., Wilson, C. M. & Gibson, D. G. *Vibrio natriegens* as a fast-growing host for molecular biology. *Nat. Methods* **13**, 849-851 (2016).
24. Jakes, K. S. The colicin E1 TolC box: identification of a domain required for colicin E1 cytotoxicity and TolC binding. *J. Bacteriol.* **199**, e00412-16 (2017).
25. Dalia, T. N. *et al*. Multiplex genome editing by natural transformation (MuGENT) for synthetic biology in *Vibrio natriegens*. *ACS Synth. Biol.* **6**, 1650-1655 (2017).
