# Supplementary material for: Discovery and remodeling of Vibrio natriegens as a microbial platform for efficient formic acid biorefinery
Source: Nat Commun. 2023 Nov 27;14:7758. doi: 10.1038/s41467-023-43631-2 (PMC10682008; doi:10.1038/s41467-023-43631-2)
Supplement: Supplementary file 4 — Reporting Summary [file 41467_2023_43631_MOESM4_ESM.pdf]

Corresponding author(s): Yang Gu
Last updated by author(s): Nov 7, 2023

Reporting Summary

Nature Portfolio wishes to improve the reproducibility of the work that we publish. This form provides structure for consistency and transparency in reporting. For further information on Nature Portfolio policies, see our Editorial Policies and the Editorial Policy Checklist.

Statistics

For all statistical analyses, confirm that the following items are present in the figure legend, table legend, main text, or Methods section.

n/a	Confirmed	
☐	☒	The exact sample size (*n*) for each experimental group/condition, given as a discrete number and unit of measurement
☐	☒	A statement on whether measurements were taken from distinct samples or whether the same sample was measured repeatedly
☐	☒	The statistical test(s) used AND whether they are one- or two-sided<br>*Only common tests should be described solely by name; describe more complex techniques in the Methods section.*
☒	☐	A description of all covariates tested
☒	☐	A description of any assumptions or corrections, such as tests of normality and adjustment for multiple comparisons
☐	☒	A full description of the statistical parameters including central tendency (e.g. means) or other basic estimates (e.g. regression coefficient) AND variation (e.g. standard deviation) or associated estimates of uncertainty (e.g. confidence intervals)
☐	☒	For null hypothesis testing, the test statistic (e.g. *F*, *t*, *r*) with confidence intervals, effect sizes, degrees of freedom and *P* value noted<br>*Give P values as exact values whenever suitable.*
☒	☐	For Bayesian analysis, information on the choice of priors and Markov chain Monte Carlo settings
☒	☐	For hierarchical and complex designs, identification of the appropriate level for tests and full reporting of outcomes
☒	☐	Estimates of effect sizes (e.g. Cohen's *d*, Pearson's *r*), indicating how they were calculated

Our web collection on statistics for biologists contains articles on many of the points above.

Software and code

Policy information about availability of computer code

Data collection	LC-MS data were collected by Q Exactive quadrupole orbitrap high-resolution mass spectrometry coupled with a Dionex Ultimate 3000 RSLC (HPG) ultra-performance liquid chromatography (UPLC-Q-Orbitrap-HRMS) system (Thermo Fisher Scientific), with a HESI ionization source. GC-MS data were collected by GC-MS system (GC7890-MS7200QTOF, Agilent Tech.). qRT-PCR experiments were performed by using Bio-Rad iQ5 real-time PCR detection system (Bio-Rad, Palo Alto, USA). Genome sequencing (re-sequencing) was performed on MGISEQ-2000 (Mgi Tech Co., Ltd., Shen Zhen, China). RNA-seq data collected was completed on the Illumina HiSeq × Ten.
Data analysis	Graphpad (version 7.0) software was used to analyze the data on cell growth, production, gene expression. Mass HunterWorkstation (version B.08.00) was used for the analysis of LC-MS data. GC-MS system (GC7890-MS7200QTOF, Agilent Tech.) was used for the analysis of GC-MS data. The online platform of Majorbio Cloud Platform (www.majorbio.com) was used for RNA-seq bioinformatics analyses. Bio-Rad iQ5 real-time PCR detection system (Bio-Rad, Palo Alto, USA) was used to analyze RT-PCR results. BWA (version 0.7.10) and GATK (version 3.4.0) were used for genome sequencing (re-sequencing) analysis.

For manuscripts utilizing custom algorithms or software that are central to the research but not yet described in published literature, software must be made available to editors and reviewers. We strongly encourage code deposition in a community repository (e.g. GitHub). See the Nature Portfolio guidelines for submitting code & software for further information.

Data

Policy information about availability of data

All manuscripts must include a data availability statement. This statement should provide the following information, where applicable:

- Accession codes, unique identifiers, or web links for publicly available datasets
- A description of any restrictions on data availability
- For clinical datasets or third party data, please ensure that the statement adheres to our policy

The RNA-seq data and genome resequencing data generated in this study have been deposited in the NCBI SRA database under bioproject ID PRJNA843433 and PRJNA1022572, respectively. The data supporting the findings of this study are available in the text and the supplementary figures and tables or can be obtained from the corresponding authors upon reasonable requests. Source data are provided with this paper.

Human research participants

Policy information about studies involving human research participants and Sex and Gender in Research.

Reporting on sex and gender	Not applicable for this study.
Population characteristics	Not applicable for this study.
Recruitment	Not applicable for this study.
Ethics oversight	Not applicable for this study.

Note that full information on the approval of the study protocol must also be provided in the manuscript.

Field-specific reporting

Please select the one below that is the best fit for your research. If you are not sure, read the appropriate sections before making your selection.

☒ Life sciences ☐ Behavioural & social sciences ☐ Ecological, evolutionary & environmental sciences

For a reference copy of the document with all sections, see nature.com/documents/nr-reporting-summary-flat.pdf

Life sciences study design

All studies must disclose on these points even when the disclosure is negative.

Sample size	Samples size for each experiment is indicated in figure legends. No statistical methods were used to predetermine sample size. The samples size (triplicates) for microbial fermentations was chosen based on the common approach in this field.
Data exclusions	No data were excluded.
Replication	All experiments were successfully repeated at least twice, and the number of independent experiments or biological replicates is indicated in the figure legends. The Isotopic tracing experiment was performed without repeating. For all data showing representative chromatograms and mass spectra, at least three biological replicates were performed.
Randomization	Samples were randomly assigned to the treatment and control groups with no formal randomization techniques.
Blinding	This work is focused on microbial modifications, laboratory adaptive evolution, and fermentation, and blinding is not relevant to these experiments.

Reporting for specific materials, systems and methods

We require information from authors about some types of materials, experimental systems and methods used in many studies. Here, indicate whether each material, system or method listed is relevant to your study. If you are not sure if a list item applies to your research, read the appropriate section before selecting a response.

Materials & experimental systems

n/a	Involved in the study
☒	☐ Antibodies
☒	☐ Eukaryotic cell lines
☒	☐ Palaeontology and archaeology
☒	☐ Animals and other organisms
☒	☐ Clinical data
☒	☐ Dual use research of concern

Methods

n/a	Involved in the study
☒	☐ ChIP-seq
☒	☐ Flow cytometry
☒	☐ MRI-based neuroimaging
