# Supplementary material for: Discovery and remodeling of Vibrio natriegens as a microbial platform for efficient formic acid biorefinery
Source: Nat Commun. 2023 Nov 27;14:7758. doi: 10.1038/s41467-023-43631-2 (PMC10682008; doi:10.1038/s41467-023-43631-2)
Supplement: Supplementary file 5 — Supplementary Table [file 41467_2023_43631_MOESM5_ESM.doc]

**Supplementary Table 5:** The primers used in this work

| **Names** | **Sequences (5'→3')** | **Description** |
| --- | --- | --- |
| Cm_F | GAAAGATTAGCGATTGTCGCGATTGGTGAGGATTAAAATACCTGTGACGGAAGATCAC | Forward primer for cloning the chloramphenicol resistance gene |
| Cm_R | TTTAAAGACTTTAACTATGAAATACCTGTTCTCTGTCGAATTTGCTTTCGAATTTCTGC | Reverse primer for cloning the chloramphenicol resistance gene |
| Kan_F | ATTGTCGCGATTGGTGAGGATTATTTGTTATCATTCTATAGTATTAAGTATTGTTCAGC | Forward primer for cloning the kanamycin resistance gene |
| Kan_R | CTTTTTAAAGACTTTAACTATGAAATACCTGTTCTCTAGTTCCTGCCCTCTGATTTTCC | Reverse primer for cloning the kanamycin resistance gene |
| KO_dns_UP_F | TAATCCTCACCAATCGCGAC | Forward primer for cloning the upstream homologous arm of the *dns* gene |
| KO_dns_UP_R | ACTGGTAAGCCATAACGACC | Reverse primer for cloning the upstream homologous arm of the *dns* gene |
| KO_dns_DN_F | CTAACATGGCTAAGCACCTG | Forward primer for cloning the downstream homologous arm of the *dns* gene |
| KO_dns_DN_R | AGAGAACAGGTATTTCATAGTTAAAGTC | Reverse primer for cloning the downstream homologous arm of the *dns* gene |
| KO_00930_DN_F | GGAAATTCCCTGCCTTTATTGTTTCTGTGCTTCTTTTTCTCGTCCTTAAAATGGAAAGG | Forward primer for cloning the downstream homologous arm of the *PN96_00930* gene |
| KO_00930_DN_R | GAACAATCTGTAATTAGTGGATCGTTG | Reverse primer for cloning the downstream homologous arm of the *PN96_00930* gene |
| KO_00930_UP_F | CACTAGAAAAAGATCCACGCATCC | Forward primer for cloning the upstream homologous arm of the *PN96_00930* gene |
| KO_00930_UP_R | GAAGCACAGAAACAATAAAGGCAG | Reverse primer for cloning the upstream homologous arm of the *PN96_00930* gene |
| KO_00930_seq_F | GGTGAGTAATCAGGCTGAGTATC | Forward primer for identifying the *PN96_00930* knockout |
| KO_00930_seq_R | CTAATGGTCTGTTTGCTCATCGTG | Reverse primer for identifying the *PN96_00930* knockout |
| KO_14755_DN_F | AAAATAAAAAGAGAAACGCCCACGACAAAGATTTAAATGACATAAAAAAATGGGACGCG | Forward primer for cloning the downstream homologous arm of the *PN96_14755* gene |
| KO_14755_DN_R | CTACGGCAGCAATCAGAGAGTAAC | Reverse primer for cloning the downstream homologous arm of the *PN96_14755* gene |
| KO_14755_UP_F | ACTCAAGTGAGATGGCGTTTAAG | Forward primer for cloning the upstream homologous arm of the *PN96_14755* gene |
| KO_14755_UP_R | GTGGGCGTTTCTCTTTTTATTTTTTAG | Reverse primer for cloning the upstream homologous arm of the *PN96_14755* gene |
| KO_14755_seq_F | CCGATCATCTCATTGAACGTATTC | Forward primer for identifying the *PN96_14755* knockout |
| KO_14755_seq_R | GTAAAGATCGCCCTGACGATATTC | Reverse primer for identifying the *PN96_14755* knockout |
| KO_07295_DN_F | TGGCGCGCATCCTTCAACCCTACATAAGGCTCCAAATTTAACCGCAAAAGAAAAACTCG | Forward primer for cloning the downstream homologous arm of the *PN96_07295* gene |
| KO_07295_DN_R | ACCATACAGTTTTCTTCGCCC | Reverse primer for cloning the downstream homologous arm of the *PN96_07295* gene |
| KO_07295_UP_F | CAAAGATGGGGGCGATGGTG | Forward primer for cloning the upstream homologous arm of the *PN96_07295* gene |
| KO_07295_UP_R | TTGGAGCCTTATGTAGGGTTGAAG | Reverse primer for cloning the upstream homologous arm of the *PN96_07295* gene |
| KO_07295_seq_F | GAGAATCACGCGACTTTGAAAC | Forward primer for identifying the *PN96_07295* knockout |
| KO_07295_seq_R | CTCATCATATCTGATACAAACAAAAAG | Reverse primer for identifying the *PN96_07295* knockout |
| KO_10585_DN_F | CAAGATTGATAAAGCACGTTAAGGATGAATGATTGAATACGAATACCAGAAAACAATAC | Forward primer for cloning the downstream homologous arm of the *PN96_10585* gene |
| KO_10585_DN_R | CAAAAAGCCAATCGCCGTC | Reverse primer for cloning the downstream homologous arm of the *PN96_10585* gene |
| KO_10585_UP_F | CTCCAGAGCTTGCTCTGCGTTAC | Forward primer for cloning the upstream homologous arm of the *PN96_10585* gene |
| KO_10585_UP_R | AATCATTCATCCTTAACGTGCTTTATC | Reverse primer for cloning the upstream homologous arm of the *PN96_10585* gene |
| KO_10585_seq_R | GCTGTGCAATTGTGTTTGCAG | Forward primer for identifying the *PN96_10585* knockout |
| KO_10585_seq_F | CATCAAAAATCAACCGTTTGATCCG | Reverse primer for identifying the *PN96_10585* knockout |
| KO_06470_DN_F | CTTTCGACTACTTGTTATTTTAGAGAGAGATTACACCCCATCTCCTGCGGTTTTCAACG | Forward primer for cloning the downstream homologous arm of the *PN96_06470* gene |
| KO_06470_DN_R | ATTTGAACGGGTAATATAGTTTGCC | Reverse primer for cloning the downstream homologous arm of the *PN96_06470* gene |
| KO_06470_UP_F | ATTCACTAACCCCCGAAAAAGTAG | Forward primer for cloning the upstream homologous arm of the *PN96_06470* gene |
| KO_06470_UP_R | TGGGGTGTAATCTCTCTCTAAAATAAC | Reverse primer for cloning the upstream homologous arm of the *PN96_06470* gene |
| KO_06470_seq_F | GCTCTCTAACAAATGGGCTTTG | Forward primer for identifying the *PN96_06470* knockout |
| KO_06470_seq_R | CCACAAGAGCATTTGGGTTTG | Reverse primer for identifying the *PN96_06470* knockout |
| KO_19465_DN_F | CAACTACTTGTTATTTTAGAGAGATTACACCCCACGCCTTACGGTTTTGGTCGTTATAC | Forward primer for cloning the downstream homologous arm of the *PN96_19465* gene |
| KO_19465_DN_R | AAGTAAACAAAGCGGTATAGACATTAG | Reverse primer for cloning the downstream homologous arm of the *PN96_19465* gene |
| KO_19465_UP_F | TTTCTTGCCGTTTCATGGACAG | Forward primer for cloning the upstream homologous arm of the *PN96_19465* gene |
| KO_19465_UP_R | TGGGGTGTAATCTCTCTAAAATAACAAG | Reverse primer for cloning the upstream homologous arm of the *PN96_19465* gene |
| KO_19465_seq_F | GCAATTTTAACAATAAGAACAAACACG | Forward primer for identifying the *PN96_19465* knockout |
| KO_19465_seq_R | CAAAAAAGTTATCGCGTCTAACG | Reverse primer for identifying the *PN96_19465* knockout |
| KO_11695_DN_F | GTTTAAGCGTTTATACGTTTATAAAAAGC | Forward primer for cloning the downstream homologous arm of the *PN96_11695* gene |
| KO_11695_DN_R | CAGCAGATACAGCGCGAATAAAG | Reverse primer for cloning the downstream homologous arm of the *PN96_11695* gene |
| KO_11695_UP_F | TAGATATAAGACGCTGAAAAGCTACC | Forward primer for cloning the upstream homologous arm of the *PN96_11695* gene |
| KO_11695_UP_R | GTAGTTCTCCTTGAGAGTATTTTTTTATAAATG | Reverse primer for cloning the upstream homologous arm of the *PN96_11695* gene |
| KO_11695_kanR_F | AAAATACTCTCAAGGAGAACTACTTTGTTATCATTCTATAGTATTAAGTATTGTTCAGC | Forward primer for cloning the kanamycin resistance gene |
| KO_11695_kanR_R | TAAGATCGGCTTTTTATAAACGTATAAACGCTTAAACAGTTCCTGCCCTCTGATTTTCC | Reverse primer for cloning the kanamycin resistance gene |
| KO_11695_seq_F | GTGATTCCTATAGCTTTACTTGGTTG | Forward primer for identifying the *PN96_11695* knockout |
| KO_11695_seq_R | CGAAGCGATCGAGAAAGCAAAC | Reverse primer for identifying the *PN96_11695* knockout |
| KO_20840_DN_F | ACGGTTTAGTCACTTTGGGAGCATCCCATCACATCCTCCTGTCATTTAAATTAAACTTC | Forward primer for cloning the downstream homologous arm of the *PN96_20840* gene |
| KO_20840_DN_R | CGGAGCTTGCTCAGTGGAATG | Reverse primer for cloning the downstream homologous arm of the *PN96_20840* gene |
| KO_20840_UP_F | GATGTTCGATATATTGCGAGAGCC | Forward primer for cloning the upstream homologous arm of the *PN96_20840* gene |
| KO_20840_UP_R | GGATGCTCCCAAAGTGACTAAAC | Reverse primer for cloning the upstream homologous arm of the *PN96_20840* gene |
| KO_20840_seq_F | GCGAAGACAGTTTAGAAGAGGC | Forward primer for identifying the *PN96_20840* knockout |
| KO_20840_seq_R | CAATGTAAAGTAATGCCGAAACAATG | Reverse primer for identifying the *PN96_20840* knockout |
| KO_08455_DN_R | GCATTACTTGGATCGAGTTGAGAC | Forward primer for cloning the downstream homologous arm of the *PN96_08455* gene |
| KO_08455_DN_F | TACGTATTTTTTTCTACTAAAAAGGTAGGTATGTCGACTGTCGCGAAATAACGTTATAG | Reverse primer for cloning the downstream homologous arm of the *PN96_08455* gene |
| KO_08455_UP_R | GACATACCTACCTTTTTAGTAGAAAAAAATACG | Forward primer for cloning the upstream homologous arm of the *PN96_08455* gene |
| KO_08455_UP_F | CGGATGGCGTAGATGACGAAC | Reverse primer for cloning the upstream homologous arm of thePN96_08455 gene |
| KO_08455_seq_F | CTTGTCGCTACCCCATTTTTATC | Forward primer for identifying the *PN96_08455* knockout |
| KO_08455_Seq_R | CGTTGGTGTAATCATGGTCAAC | Reverse primer for identifying the *PN96_08455* knockout |
| KO_05880_DN_F | GTGAGCAAGTAGCTCCTTCACTGAACAGGACAAGACTTTTGCAAAAGCAAAGTTTGGTG | Forward primer for cloning the downstream homologous arm of the *PN96_05880* gene |
| KO_05880_DN_R | GTATCGAGGTTCGCGTGATTTATTC | Reverse primer for cloning the downstream homologous arm of the *PN96_05880* gene |
| KO_05880_UP_F | CAAATTTAAGAGACGGAGACTGACC | Forward primer for cloning the upstream homologous arm of the *PN96_05880* gene |
| KO_05880_UP_R | AGTCTTGTCCTGTTCAGTGAAGG | Reverse primer for cloning the upstream homologous arm of the *PN96_05880* gene |
| KO_05880_seq_F | GTATTAAGTAGCGTGAATCTCTCGC | Forward primer for identifying the *PN96_05880* knockout |
| KO_05880_seq_R | CCTTTGTGAATAAACGGTATTAGCG | Reverse primer for identifying the *PN96_05880* knockout |
| KO_05840_50_UP_F | GTTCTTGCGACTAAGCGCATTTTC | Forward primer for cloning the downstream homologous arm of the *PN96_05840*, *PN96_05845*, *PN96_05850* genes |
| KO_05840_50_UP_R | CGGGATTTGGATTGCGTACATC | Reverse primer for cloning the downstream homologous arm of the *PN96_05840*, *PN96_05845*, *PN96_05850* genes |
| KO_05850_50_DN_F | GTAAAACAACTCAGGATGTACGCAATCCAAATCCCGCTCTTACCTCCTAAAGTGTGTCG | Forward primer for cloning the upstream homologous arm of the *PN96_05840*, *PN96_05845*, *PN96_05850* genes |
| KO_05850_50_DN_R | CAAGAAATCGTCGAACCTGCTC | Reverse primer for cloning the upstream homologous arm of the *PN96_05840*, *PN96_05845*, *PN96_05850* genes |
| KO_05850_50_Seq_F | CCAAACGATAACCAGCATAAATAATAGC | Forward primer for identifying the knockout of *PN96_05840*, *PN96_05845* and *PN96_05850* |
| KO_05850_50_seq_R | CAGGAACTACGAAAGTGGCAAAC | Reverse primer for identifying the knock of *PN96_05840*, *PN96_05845* and *PN96_05850* |
| KO_21155_DN_F | AGTTCATCTAAAGAATCTAACAGGTAAGTCTTTGATGATTGAGAAGTGGAATTGATGGC | Forward primer for cloning the downstream homologous arm of the *PN96_21155* gene |
| KO_21155_DN_R | GATTACATAGATAGCAGACTGCTTTTTATTTTAC | Reverse primer for cloning the downstream homologous arm of the *PN96_21155* gene |
| KO_21155_UP_F | CACCGATCCGGACTGGGTGC | Forward primer for cloning the upstream homologous arm of the *PN96_21155* gene |
| KO_21155_UP_R | CAAAGACTTACCTGTTAGATTCTTTAG | Reverse primer for cloning the upstream homologous arm of the *PN96_21155* gene |
| KO_21155_Seq_F | GAAGAACCCCTTAGCCGAATG | Forward primer for identifying the *PN96_21155* knockout |
| KO_21155_Seq_R | CAACGGGTAACTGTGGACTG | Reverse primer for identifying the *PN96_21155* knockout |
| KO_22795_DN_F | TTCGAGGTTCAATGCGAAGCCCTTTTTATCCTGTCTGCACGCTACCCTGAGTTAGCCAC | Forward primer for cloning the downstream homologous arm of the *PN96_22795* gene |
| KO_22795_DN_R | CGTAATCAACCGTACCTTGCC | Reverse primer for cloning the downstream homologous arm of the *PN96_22795* gene |
| KO_22795_UP_F | CAGCGAAAGCAGATGTTGCG | Forward primer for cloning the upstream homologous arm of the *PN96_22795* gene |
| KO_22795_UP_R | GCAGACAGGATAAAAAGGGC | Reverse primer for cloning the upstream homologous arm of the *PN96_22795* gene |
| KO_22795_seq_F | CTGAATAACAGAGGGTCGTCAG | Forward primer for identifying the *PN96_22795* knockout |
| KO_22795_seq_R | GTTAGTGAGCATTTTGTCACATGAAC | Reverse primer for identifying the *PN96_22795* knockout |
| tfox_F | AAAGAAAATGCCGATACTCGAGCTTCTGCTCCCGA | Forward primer for cloning the DNA fragment of *tfox* |
| tfox_R | TCTGGCCTATGGAGCTGTGCGGCAGCGCTCAGTAGG | Reverse primer for cloning the DNA fragment of *tfox* |
| sacB_F | GCTCCATAGGCCAGATCTTTAGGCCCGTAGTCTGCAA | Forward primer for cloning the DNA fragment of *sacB* |
| sacB_R | GGAGCAGAAGCTCGAGTATCGGCATTTTCTTTTGCGT | Reverse primer for cloning the DNA fragment of *sacB* |
| pColE1-AmpR-F | TCTAGAGGATCCCCGGGTAC | Forward primer for cloning the linear plasmid pColE1-Amp vector |
| pColE1-AmpR-R | GAGGATATCACAATGGTGTCGTACGAAGAGCTTTTATAGCATGTGAGCAAAAGGCCAGC | Reverse primer for cloning the linear plasmid pColE1-Amp vector |
| idgs-sfp-F | ATTTTGTAGAGTCACACAGGAAAGTACTAATACCATGATGACCCTTCAAGAAACCAGCG | Forward primer for cloning the DNA fragment of idgs-sfp |
| idgs-sfp-R | CTATAAAAGCTCTTCGTACGACACC | Reverse primer for cloning the DNA fragment of idgs-sfp |
| J23102-B0032m-F | GAATTCGAGCTCGGTACCCGGGGATCCTCTAGATTGACAGCTAGCTCAGTCCTAGGTACTGTGC | Forward primer for cloning the DNA fragment of J23102-B0032m |
| J23102-B0032m-R | TTAGTACTTTCCTGTGTGACTCTACAAAATTATTGCTAGCACAGTACCTAGGACTGAGC | Reverse primer for cloning the DNA fragment of J23102-B0032m |
| RT00600-F | ACTGGCGACATCTTGATTGGT | Forward primer for *PN96_00600* RT-PCR |
| RT00600-R | AAAGCGGGAACACTTGTCCA | Reverse primer for *PN96_00600* RT-PCR |
| RT04975-F | TATCCCGGTAGAGGCTCTGG | Forward primer for *PN96_04975* RT-PCR |
| RT04975-R | CCCTGCCGTTTCTACCACAT | Reverse primer for *PN96_04975* RT-PCR |
| RT05880-F | CCCATTCTGCCGCAGTGATA | Forward primer for *PN96_05880* RT-PCR |
| RT05880-R | GACTGCTTAAGCGGCACTTG | Reverse primer for *PN96_05880* RT-PCR |
| RT00275-F | AAACGTTGTTCGTGCTGGTG | Forward primer for *PN96_00275* RT-PCR |
| RT00275-R | TGACGTAACTGCGTCGACAA | Reverse primer for *PN96_00275* RT-PCR |
| RT01795-F | AGGGGCAATGTTGACCTACG | Forward primer for *PN96_01795* RT-PCR |
| RT01795-R | CGTTCAGTAATGTGCGCGAG | Reverse primer for *PN96_01795* RT-PCR |
| RT00890-F | CTGATCGCGTTCTGGGATGA | Forward primer for *PN96_00890* RT-PCR |
| RT00890-R | CACCGTGACAGTCGTGAGAA | Reverse primer for *PN96_00890* RT-PCR |
| RT01355-F | TTCAAAGGTGCTGGCTGGAA | Forward primer for *PN96_01355* RT-PCR |
| RT01355-R | ACCACGCTTAAGTGCGAAGA | Reverse primer for *PN96_01355* RT-PCR |
| RT01430-F | TCGAAACTGAACATGGGCGA | Forward primer for *PN96_01430* RT-PCR |
| RT01430-R | CAGCAACGTCACCTGGTTTG | Reverse primer for *PN96_01430* RT-PCR |
| RT04350-F | TGACGTTATCGCGTTCACCA | Forward primer for *PN96_04350* RT-PCR |
| RT04350-R | CCATCTCACCAAGCGCCTTA | Reverse primer for *PN96_04350* RT-PCR |
| RT03705-F | TGAGCAGCACCCTAGAAACG | Forward primer for *PN96_03705* RT-PCR |
| RT03705-R | ACTCAATTCACCGCTCTGGG | Reverse primer for *PN96_03705* RT-PCR |
| RT03730-F | TGCCAAGGGATACCGACAAC | Forward primer for *PN96_03730* RT-PCR |
| RT03730-R | GGCCCAGTTGTGAGGCTTTA | Reverse primer for *PN96_03730* RT-PCR |
| RT00040-F | ACAACGCGGTTTGATTGGTG | Forward primer for *PN96_00040* RT-PCR |
| RT00040-R | AATTGCGCCATTGAGTCTGC | Reverse primer for *PN96_00040* RT-PCR |
| RT00085-F | CTGCAACAGATGGCCCAATG | Forward primer for *PN96_00085* RT-PCR |
| RT00085-R | GCGCTTCTGCTAGTTCAACG | Reverse primer for *PN96_00085* RT-PCR |
| RT00580-F | TTCGCTCGTACTTTACGCGA | Forward primer for *PN96_00580* RT-PCR |
| RT00580-R | CTAGAGGGCGACGCATCTTC | Reverse primer for *PN96_00580* RT-PCR |
| RT00205-R | GTTAGCGACAGAAGAAGCAC | Forward primer for *PN96_00205* RT-PCR |
| RT00205-R | CCGGTATTCCTTCAGATCTC | Reverse primer for *PN96_00205* RT-PCR |
| RT01345-R | ATACGTGGGTCTTCATGCGG | Reverse primer for *PN96_01345* RT-PCR |
